# High dose multiple micronutrient supplementation improves villous morphology in environmental enteropathy without HIV enteropathy: results from a double-blind randomised placebo controlled trial in Zambian adults

**DOI:** 10.1186/1471-230X-14-15

**Published:** 2014-01-15

**Authors:** John Louis-Auguste, Stephen Greenwald, Michelo Simuyandi, Rose Soko, Rose Banda, Paul Kelly

**Affiliations:** 1 Blizard Institute, Barts & The London School of Medicine, London, UK; 2 Tropical Gastroenterology and Nutrition Group, University of Zambia School of Medicine, Lusaka, Zambia

**Keywords:** Enteropathy, Micronutrients, Nutrition, HIV-related gastrointestinal disease, Histopathology

## Abstract

**Background:**

Environmental enteropathy (EE) is an asymptomatic abnormality of small bowel structure and function, which may underlie vaccine inefficacy in the developing world. HIV infection co-exists in many of these populations. There is currently no effective treatment. We conducted a secondary analysis of a randomised controlled trial of high dose multiple micronutrient (MM) supplementation on small bowel architecture in EE in participants with or without HIV infection.

**Methods:**

In a double-blind parallel-group trial of the effect of MM on innate immune responses to oral vaccines, consenting Zambian adults were randomised to receive 6 weeks of 24 micronutrients as a daily capsule or placebo. HIV status was established after randomisation. Proximal jejunal biopsies were obtained after the supplementation period. Villous height, crypt depth, villous width, villous perimeter per 100 μm muscularis mucosa (a measure of epithelial surface area), and villous cross sectional area per 100 μm muscularis mucosa (a measure of villous compartment volume) were measured in orientated biopsy sections using semi-automated image analysis. Analysis was by intention to treat.

**Results:**

18 patients received MM and 20 placebo. 6/18 MM and 9/20 placebo patients had HIV. In HIV negative patients given MM compared to placebo, mean villous height was 24.0% greater (293.3 v. 236.6 μm; 95% CI of difference 17.7–95.9 μm; P = 0.006), mean villous area was 27.6% greater (27623 v. 21650 μm^2^/100 μm; 95% CI of difference 818–11130 μm^2^/100 μm; P = 0.03), and median villous perimeter was 29.7% greater (355.0 v. 273.7 μm/100 μm; 95% CI of difference 16.3–146.2 μm/100 μm; P = 0.003). There was no significant effect on crypt depth or villous width. No effect was observed in HIV positive patients. There were no adverse events attributable to MM.

**Conclusions:**

MM improved small bowel villous height and absorptive area, but not crypt depth, in adults with EE without HIV. Nutritional intervention may therefore selectively influence villous compartment remodelling. In this small study, there was a clear difference in response depending on HIV status, suggesting that EE with superimposed HIV enteropathy may be a distinct pathophysiological condition.

## Background

Environmental enteropathy (EE) is an asymptomatic disorder which is highly prevalent throughout the developing world. It is thought to be the result of recurrent exposure to gastrointestinal pathogens [1], but could be explained by other factors including nutrient deficiencies. It is characterised by abnormal villous and crypt architecture, heightened T cell-mediated inflammation and increased epithelial permeability secondary to impaired tight junction function [2,3] which results in reduced small bowel absorptive capacity, increased microbial translocation and systemic inflammation [4]. This predisposes to poor growth in children [4] and malabsorption in people of all ages. Furthermore, although live oral vaccines against a number of enteric viral and bacterial pathogens including rotavirus and *Salmonella* Typhi/Paratyphi provide high rates of effective immunity in Western populations, their efficacy in low income countries is markedly reduced [5] which compromises their potential as effective public health interventions. Populations with reduced vaccine efficacy have a high prevalence of environmental enteropathy (EE) [2,6]. From an immunological perspective, chronic immune stimulation can lead to suppressed immune response to pathogenic bacteria and may therefore explain the reduced immunogenicity of oral vaccines in these populations. EE is therefore increasingly recognised as being of critical importance in global health [1].

HIV co-exists with EE in many populations, particularly in sub-Saharan Africa. We have observed that this results in an asymptomatic HIV-associated enteropathy that has subtle histological and functional differences from EE in HIV negative patients. For example compared to HIV negative patients, EE with HIV is characterised by more marked crypt hypertrophy and intestinal permeability. Villous height is also correlated with CD4 count [2,6]. This is distinct from the 'HIV enteropathy' used by some authorities to refer to a clinical condition seen in advanced HIV where no causative pathogen is readily identified, characterised by persistent diarrhoea, more severe morphological changes and marked malabsorption. The intestinal mucosal immune system is affected early and significantly in HIV infection [7], and effector T cell dysfunction may explain the exaggerated inflammation and impaired gut barrier function seen in early asymptomatic disease [2].

There is no established therapy to reverse the changes of EE. Antibiotics [8], probiotics [9], glutamine supplementation [10] and long chain fatty acid supplements [11] have been tried without success. Multiple micronutrient (MM) supplementation is an attractive potential therapy as micronutrients such as zinc [12,13] and vitamin A [14] have previously been shown to reduce morbidity and mortality from infectious diarrhoeal illnesses, hinting at an immunological role in the intestine. Furthermore, populations in which EE is prevalent also have a high prevalence of micronutrient deficiencies, which is also seen in HIV positive individuals. MM supplementation is also practical due to its low cost, ease of administration, tolerability and safety profile.

There are few data on MM interventions for EE, but in our own previously published trials, long-term lower-dose MM supplementation had a modest impact on diarrhoea and nutritional outcomes, and some effect on antimicrobial peptide expression [12,15]. Similarly, little is known about the role of MM supplementation in HIV patients [16], although several micronutrient deficiencies have been shown to be associated with worse outcomes [17]. Vitamin A and zinc supplementation is beneficial, at least in children [18]. Our study [12] demonstrated a reduction in HIV-related deaths with an MM supplement, and a recent trial of 3 vitamins with selenium showed a significant reduced disease progression and HIV-related death [19], again hinting at a role for enteral micronutrient supplementation in enhancing mucosal and systemic immunological function.

We have also shown that lower micronutrient doses did not reliably increase the concentrations of the micronutrients in blood [12]. While this might signify that these nutrients were being redistributed or utilised, it might also reflect the very malabsorptive problem which we are trying to overcome. We postulated that micronutrient supplementation at higher doses than used in our previous trial might improve mucosal architecture in patients with EE.

## Methods

### Trial design and study setting

This was a secondary study of endoscopic small bowel biopsies obtained during the course of a single centre, randomised, double-blind, placebo-controlled, parallel-group trial investigating the effect of high dose MM supplementation on innate immune effects of oral vaccines. This was carried out in adult volunteers living in a poor community in Lusaka, Zambia between June and October 2009, where we have demonstrated that EE is virtually universal [2].

### Participants

Volunteers were recruited from among residents of Misisi suburb, where we have carried out several previous studies of intestinal inflammation. Age between 18 and 60 years was the only inclusion criterion. The only absolute exclusion criterion was helminth infection (diagnosed by stool microscopy at screening). Participants who were pregnant, lactating, had had vaccination within 6 months, had taken antibiotics or non-steroidal anti-inflammatory drugs within two weeks, or who had had diarrhoea within one month were not included in any part of the study until their temporary exclusion criterion no longer applied. Informed consent followed a three stage process: door-to-door notification, focus group discussions, and individual counselling leading to fully informed written consent.

### Micronutrient supplementation, treatment allocation, and randomisation

The composition of the multiple micronutrient supplement (Immunace®, Vitabiotics, London, UK) is shown in Table 1, together with dosages of the supplement used in our earlier study [12]. The supplement was chosen to provide micronutrients at higher dosages than in the earlier study with the exception of iron (due to concerns of poorer outcome in malaria with iron [20]) and copper (due to theoretical concern of long-term toxicity). An indistinguishable placebo was manufactured and packaged in identical plastic light-proof bottles. The bottles were labelled only with a letter of the 4-letter code (two letters representing MM and the other two representing placebo) held by the manufacturer until morphometric analysis had been completed, when the databases had been locked. Participants were randomised to one of these 4 letters using a computer-generated sequence in a 1:1:1:1 ratio. Treatment allocation was therefore masked from participants and investigators throughout the duration of the study.

**Table 1:** **Composition of micronutrient supplement compared to Reference Nutrient Intake (RNI) for British adults (men or women, whichever is the higher) **[21]

**Micronutrient**	**Daily dose**	**Daily dose in 2008 study**	**RNI**	**Multiples of RNI in current study**
Vitamin A				
Retinyl palmitate (mg)	1.6 (4840 i.u.)	-	0.7	2.3
Betacarotene (mg)	6.0	4.8	4.2	1.4
Vitamin D (μg)	20	5	10^a^	2
Vitamin E (mg)	80	10	4	20
Vitamin K (μg)	140	-	1/kg/d	2
Vitamin C (mg)	300	70	40	7.5
B vitamins				
Thiamin (B1) (mg)	36	1.4	0.9	40
Riboflavin (B2) (mg)	12	1.4	1.3	9.2
Pyridoxine (B6) (mg)	20	1.9	1.4	14.2
Niacin (B3) (mg)	54	18	16	3.4
Folic acid (B9) (mg)	1	0.4	0.2	5
Cobalamin (B12) (μg)	28	-	1.5	18.7
Pantothenic acid (B5) (mg)	40	-	5^b^	8
Minerals				
Iron (mg)	16	30	14.8	1.08
Zinc (mg)	30	15	9.5	3.2
Copper (mg)	1	2	1.2	0.83
Selenium (μg)	350	65	75	4.7
Iodine (μg)	400	150	140	2.9
Chromium (μg)	200	-	25^b^	8
Magnesium (μg)^c^	100	-	250	0.4
Manganese (mg)	8	-	1.4^b^	5.7
L-Cystine (mg)	80	-	-	-
L-Carnitine (mg)	60	-	-	-
Citrus bioflavonoids (mg)	60	-	-	-

^a^Based on RNI for older adults; no intake of pre-formed vitamin D is required for adults exposed to sun. ^b^Not a formal RNI but probably an adequate intake. ^c^Full dose RNI of Magnesium cannot be administered in this form as it may predispose to diarrhea.

### Interventions

**Figure 1:**
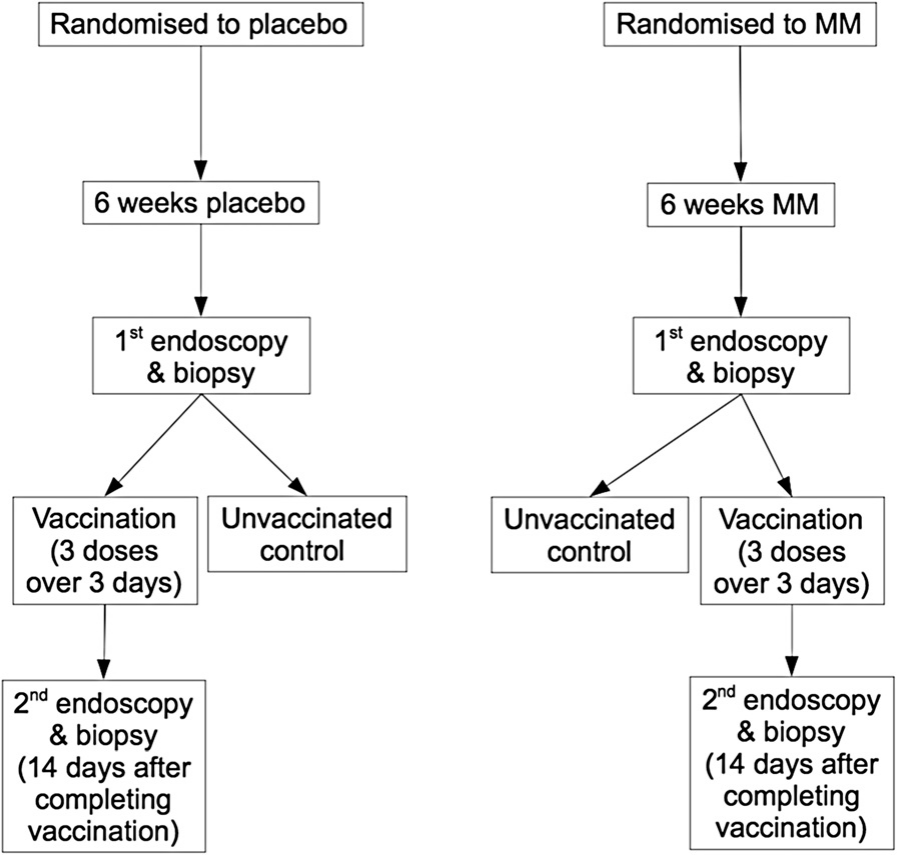
Study outline.

Participants were provided with a six week supply of once-daily trial medication and given instructions on how to take it (days 1 – 42). After the supplementation period they underwent enteroscopy with proximal jejunal biopsy under conscious sedation (performed by PK) on day 43. Patients were then vaccinated with a full course of Ty21a oral typhoid vaccine (Vivotif ®, Crucell, Baranzate, Italy; 3 doses on days 44, 46 and 48), and were re-biopsied 14 days later (Figure 1). Unvaccinated patients were also included as negative controls for analysis of vaccination effects, and only underwent endoscopic biopsy once (after the 6 week supplementation/placebo period).

Serological HIV testing was conducted on all participants prior to vaccination after appropriate counselling. Patients who were found to be HIV positive were referred for further assessment, treatment and monitoring.

Participants were interviewed at monthly intervals to check for possible side effects, illnesses, and promote compliance with trial medication. Participants were also directly asked about incidence of diarrhoea, abdominal pain, fever, loss of appetite and nausea/vomiting.

### Morphometric analysis

**Figure 2:**
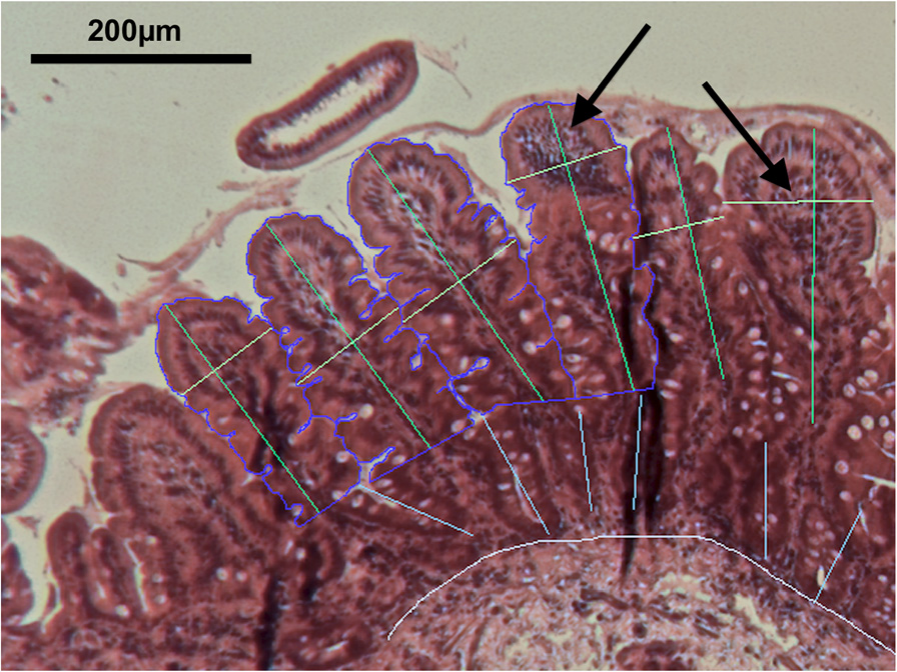
**Example of morphometric analysis.** Morphometry on a jejunal biopsy demonstrating relatively mild features of EE, with villous blunting and lymphocyte infiltration (arrows). H&E stained sections at a magnification of x8 were digitised. Automatic threshold definition was used to measure villous area (VA; area within blue lines) and villous perimeter (VP; length of blue lines excluding base). Villous height (VH; green), crypt depth (CD; cyan), maximal villous width perpendicular to VH (VW; yellow), and muscularis mucosal length (white; used as a denominator for VP and VA measurements) were drawn by eye using the cursor on the computer monitor. See also [2].

This was performed as previously described [2]. Briefly, endoscopic small intestinal biopsies were orientated under the dissecting microscope before formalin fixation and paraffin embedding. 4 μm sections were stained with haematoxylin & eosin as per standard protocols. Stained sections were first assessed for adequacy of orientation by a single investigator blinded to treatment allocation (JL-A), where both crypts and villi were seen in longitudinal section. Adequately orientated sections were then digitised using a Zeiss Axiocam digital camera system (Carl Zeiss Vision, Thornwood, NY). Images were analysed by the same investigator using a semi-automated program (written by SEG, modified from the previously described macro [2]; Additional file 1; also available at http://webspace.qmul.ac.uk/segreenwald/KS400%20macro.pdf) using a Zeiss KS400 v.33.0 image analysis system (Carl Zeiss Vision, Thornwood, NY). Maximal villous height (VH), maximal villous width perpendicular to the VH axis (VW), and crypt depth (CD) were measured, along with villous perimeter (VP) as a measure of epithelial surface area, and villous cross sectional area (VA) as a measure of villous compartment volume (Figure 2). VP and VA were expressed per 100 μm muscularis mucosae.

### Sample size, power calculations and statistical analysis

Analysis was by intention to treat. This was a secondary analysis of a study which was primarily powered to detect differences in antimicrobial peptide expression following vaccination.

Based on the morphometry results from our previous study of enteropathy in the same patient population [2], we estimated that 15 patients per treatment group would have greater than 80% power to identify a 20% difference in villous height at a 2-sided α of 0.05. We would expect a significant improvement in villous height (20% or greater) to result in clinically significant improvements in absorptive capacity and mucosal physiology. A *post hoc* power analysis in HIV positive patients showed that 6 patients would be needed to detect a difference of at least 24% (equivalent to 65 μm) in villous height (SD 36.3; 2-sided α 0.05; power 0.8).

Although VH, CD and VA were normally distributed, VP and VW were not (Shapiro-Wilk test). Two-tailed t- or paired t-tests were used for normally distributed data. Wilcoxon signed rank (for paired observations) or Kruskal-Wallis (for independent observations) tests were used for non-parametric data. Spearman correlation coefficients were used where required. Where both pre- and post-vaccination data were available, analysis was performed using either only pre- or only post-vaccination biopsy measurements. The most conservative results are presented here.

Statistics were calculated using SPSS v.21 (IBM Corporation, Armonk, New York, USA) and Stata v.12 (StataCorp LP, College Station, Texas, USA).

### Outcomes

This study was a secondary analysis. The primary comparison for this study was the difference in morphometric variables after 6 weeks of either high-dose MM supplementation or placebo depending on HIV status.

### Ethics approval and trial registration

Approval was obtained from the University of Zambia Biomedical Research Ethics Committee (007-10-07). The trial was registered as ISRCTN68751738.

## Results

### Patient groups and baseline characteristics

**Figure 3:**
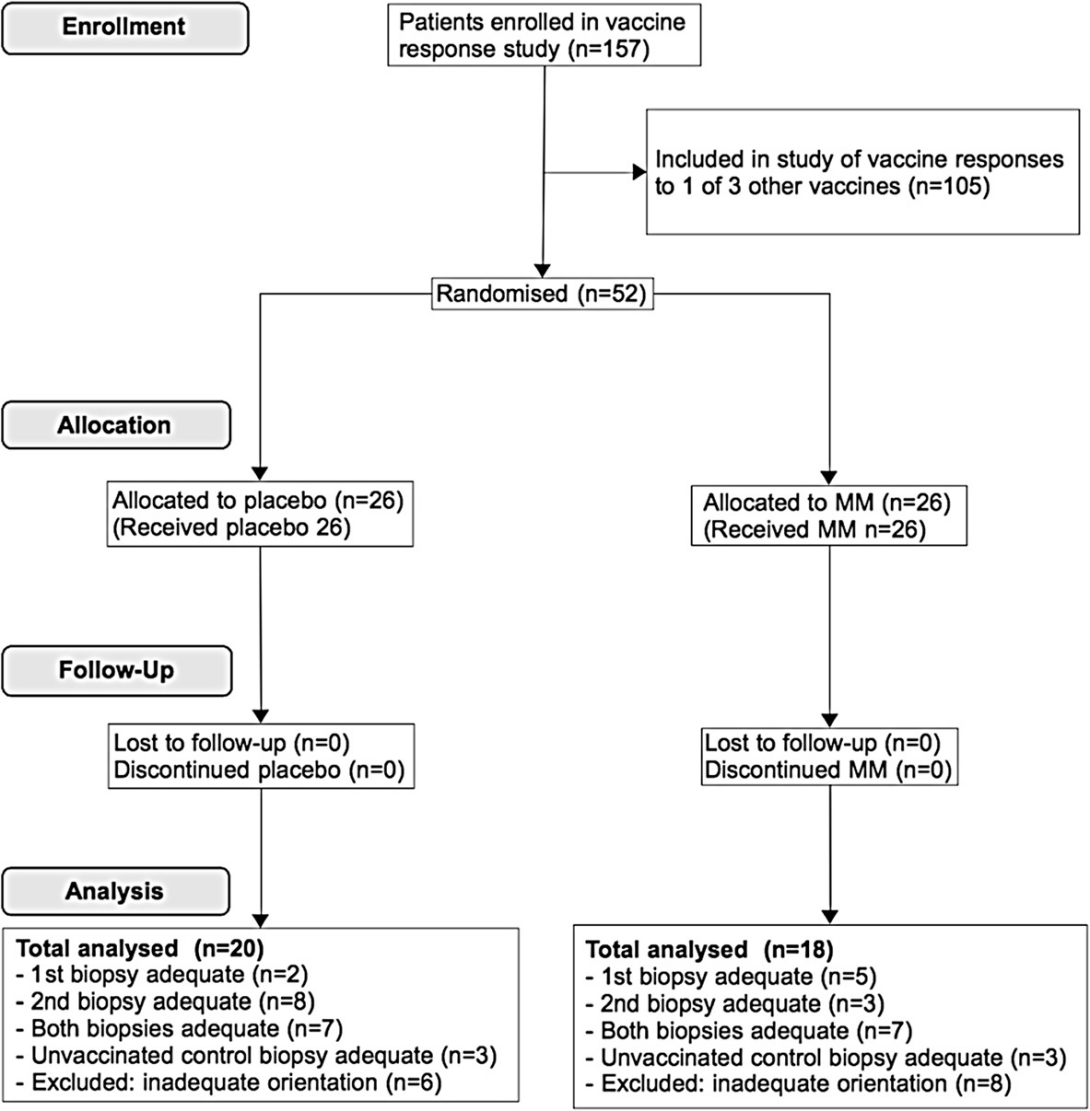
CONSORT flow diagram.

26 patients were randomised to placebo and 26 to MM supplementation. Sections suitable for morphometric analysis were available from 18/26 patients who received MM supplementation and 20/26 patients who received placebo, who all completed 6 weeks of supplement or placebo (Figure 3). 6/18 patients in the MM group and 9/20 patients in the placebo group were HIV positive (*P* = 0.52, Fisher's exact test). Baseline characteristics in MM and placebo groups were comparable (Table 2).

**Table 2:** Patient baseline characteristics

	**Placebo**	**MM**	***P***
**Number**	20	18	
**Mean (SD) age (years)**	41.8 (10.5)	35.8 (11.0)	0.1^j^
**Sex (male:female)**	16:4	11:7	0.28^k^
**HIV positive (number)**	9	6	0.52^k^
**CD4 count per μL (range)**	159-656^e^	247-959^h^	n/a
**Median (IQR) Household Hygiene Score**^**a**^	5 (4.25-9.75)	5 (4.75-5)	0.34^l^
**Never/rarely boil water (number)**	15	13	1^k^
**Never/rarely chlorinate water (number)**	11	9	1^m^
**BCG scar (number)**	16	13	0.7^k^
**Previous tuberculosis (number)**	7	4	0.49^m^
**Symptoms/signs of micronutrient deficiency**^**b**^	1	1	1^k^
**Mean (SD) BMI (kg/m**^**2**^**)**	23.7 (4.4)	24 (6.1)	0.84^j^
**Mean (SD) MUAC (cm)**	28.2 (5.5)	29.8 (6.7)	0.4^j^
**Mean (SD) fat (%)**^**c**^	37.1 (8.9)^f^	35.5 (10.5)^i^	0.64^j^
**Mean (SD) water (%)**^**c**^	46.9 (9.7)^f^	46.9 (8.2)^i^	1^j^
**Mean (SD) dynamometry (kg)**^**d**^	30.4 (6.0)^g^	31 (7.9)^i^	0.81^j^
**Never/rarely drink alcohol (number)**	14	9	0.32^m^
**Median (IQR) approx. monthly income ($)**	100 (60-120)^f^	80 (40-100)^i^	0.5^n^
**Secondary education or better (number)**	3	3	1^m^
**Electricity supply (number)**	4	5	0.71^k^

BMI, body mass index; MUAC, mid upper arm circumference. ^a^The Household Hygiene Score is an objective measure of the living conditions of each participant and ranges from 0 (worst) to 10 (best). Trained fieldworkers score up to 2 points in each of five categories (overall cleanliness; water storage facilities; food storage facilities; hand washing facilities and their use; sanitation facilities) [2]. ^b^One case of night blindness in both groups. ^c^Fat and water content was measured using a Bodystat 1500 impedance analyser (Bodystat, Douglas, Isle of Mann). ^d^Dynamometry was measured using a Takeida dynamometer. ^e^Data available in 7/9 patients. ^f^Data available in 19/20 patients. ^g^Data available in 18/20 patients. ^h^Data available in 3/6 patients. ^i^Data available in 16/18 patients. ^j^2-tailed *t* test. ^k^Fisher exact test. ^l^Chi squared test for trend. ^m^Chi squared test. ^n^Kruskal-Wallis test.

### Effect of multiple micronutrient supplementation on small bowel morphometry

In the 14 patients where both pre- and post-vaccination data were available, results of analysis using post-vaccination measurements from these 14 patients were more extreme in both magnitude and significance (Additional file 2). The more conservative results using pre-vaccination measurements from these patients are therefore presented here.

**Figure 4:**
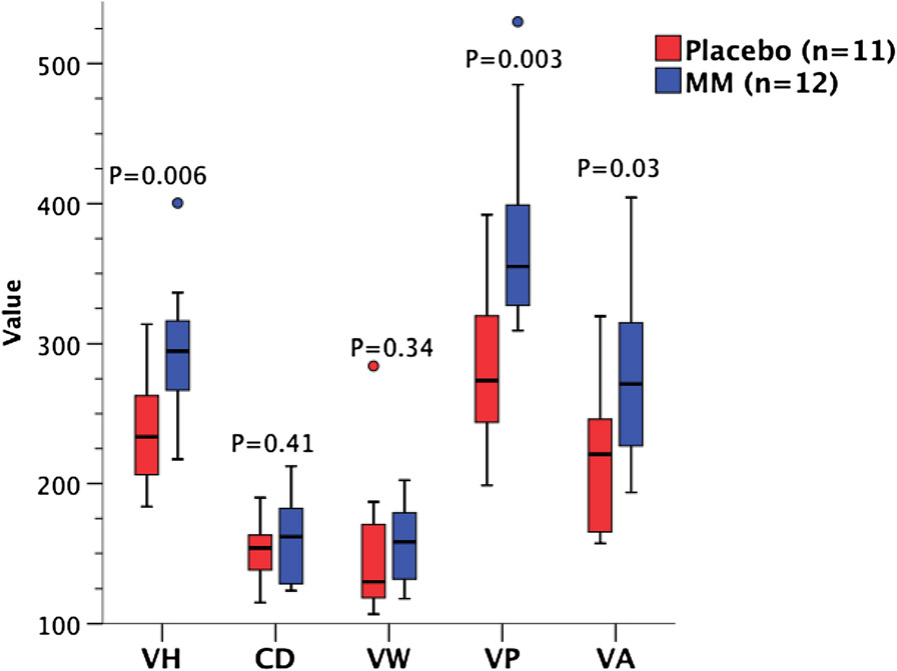
**Effect of MM in HIV negative patients.** VH, VW, CD measurements in μm; VP in μm/100 μm mucosa; VA in μm^2^/100 μm mucosa x0.01 (rescaled for ease of presentation). VH, VA and CD: 2-tailed *t* test; VW and VP: Kruskal-Wallis.

**Figure 5:**
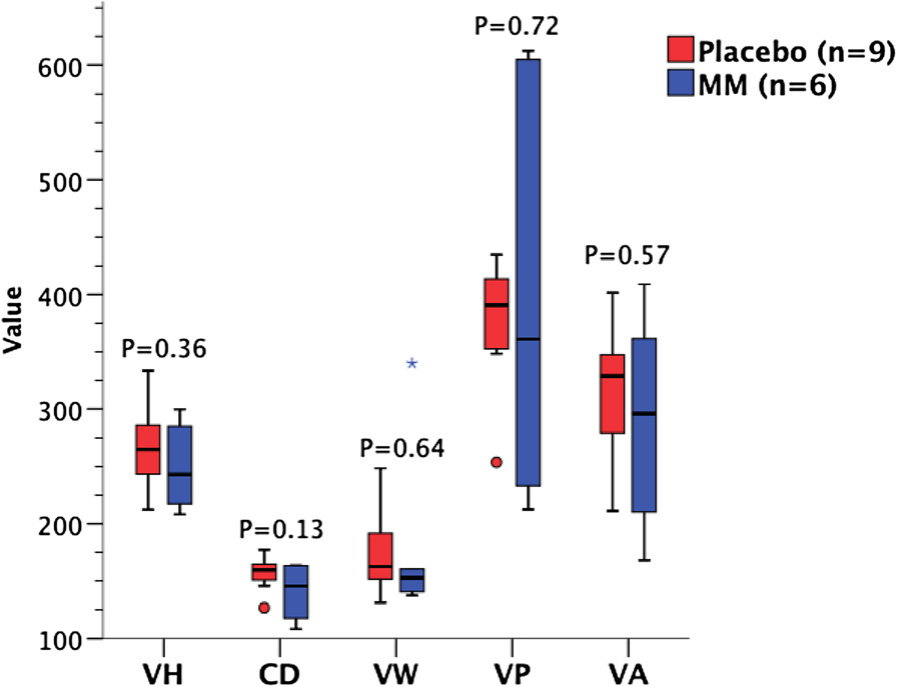
**Effect of MM in HIV positive patients.** VH, VW, CD measurements in μm; VP in μm/100 μm mucosa; VA in μm^2^/100 μm mucosa x0.01 (rescaled for ease of presentation). VH, VA and CD: 2-tailed *t* test; VW and VP: Kruskal-Wallis.

In HIV negative patients given MM compared to placebo, mean VH was 24.0% greater (293.3 v. 236.6 μm; mean difference 56.8 μm [95% confidence interval 17.7 – 95.9 μm]; *P* = 0.006, 2-tailed *t* test), mean VA per 100 μm of mucosa was 27.6% greater (27623 v. 21650 μm^2^/100 μm; mean difference 5973 μm^2^/100 μm [818 – 11130 μm^2^/100 μm]; *P* = 0.03, 2-tailed *t* test), and median VP per 100 μm of mucosa was 29.7% greater (355.0 μm/100 μm v. 273.7 μm/100 μm; median difference 81.3 μm/100 μm [16.3 – 146.2 μm/100 μm]; *P* = 0.003, Kruskal-Wallis) (Figure 4). No significant differences were observed in VW or CD. There were no changes in any morphometric variables in HIV positive patients given MM supplementation compared to placebo (Figure 5). As a result of improved VH in HIV negative patients given MM, villous:crypt (V:C) ratio was 1.56:1 with placebo v. 1.83:1 with MM (95% confidence interval of ratio following treatment 1.63:1 – 2.03:1; *P* = 0.01, 2-tailed *t* test). There was no significant difference in V:C ratio between treatment groups in HIV positive patients.

VH, VP and VA were strongly positively correlated with each other, particularly in HIV negative patients, with VP and VA having the strongest correlation. There was no significant relationship between crypt depth and any other variables in either group (Tables 3 & 4).

**Table 3:** Correlations between measured variables: HIV negative patients

	**Villous height**	**Crypt depth**	**Villous perimeter**	**Villous width**	**Villous area**
**Villous height**	1.000				
**Crypt depth**	0.18	1.000			
**Villous perimeter**	0.715^**^	0.18	1.000		
**Villous width**	0.424^*^	-0.11	0.14	1.000	
**Villous area**	0.732^**^	0.17	0.796^**^	0.426^*^	1.000

2-tailed Spearman rho. ^*^P < 0.05; ^**^P < 0.001.

**Table 4:** Correlations between measured variables: HIV positive patients

	**Villous height**	**Crypt depth**	**Villous perimeter**	**Villous width**	**Villous area**
**Villous height**	1.000				
**Crypt depth**	0.254	1.000			
**Villous perimeter**	0.514^*^	-0.236	1.000		
**Villous width**	0.182	0.182	-0.268	1.000	
**Villous area**	0.511^*^	-0.154	0.882^**^	0.093	1.000

2-tailed Spearman rho. ^*^P < 0.05; ^**^P < 0.001.

### Effect of Ty21a vaccination on morphometry

**Figure 6:**
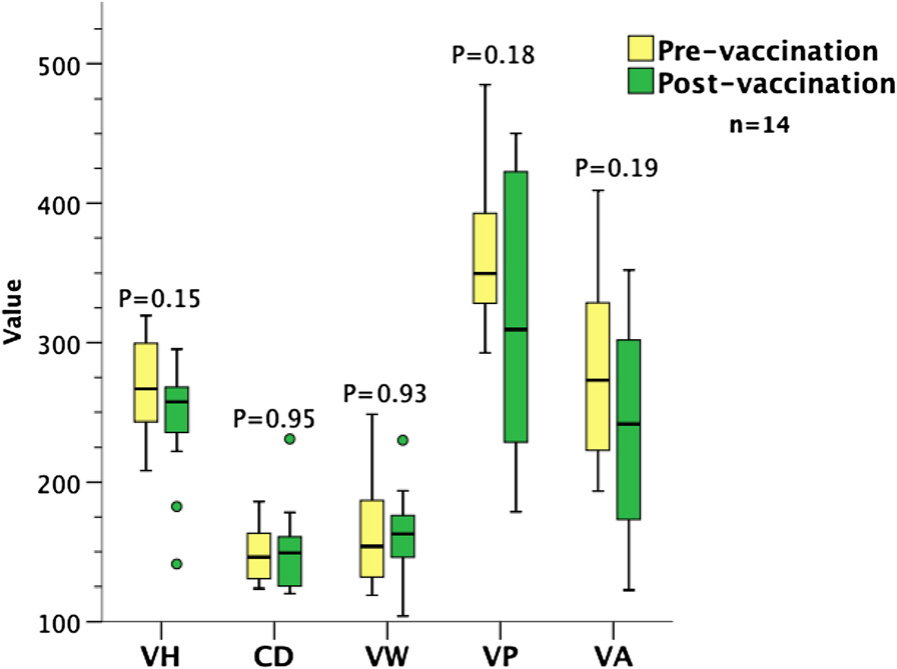
**Immunisation with Vivotif vaccine had no effect on any of the measured variables.** VH, VW, CD measurements in μm; VP in μm/100 μm mucosa; VA in μm^2^/100 μm mucosa x0.01 (rescaled for ease of presentation). n = 14. VH, VA and CD: paired 2-tailed *t* test; VW and VP: Wilcoxon signed rank.

Adequately orientated biopsies before and after the vaccination phase were available in 14 patients. This subgroup was used to exclude an effect of vaccination on small bowel architecture. In this group as a whole, vaccination had no effect on any of the variables measured (Figure 6). Furthermore, neither HIV status nor treatment allocation influenced any of the measured variables (Kruskal-Wallis).

### Safety

No serious adverse events were reported over the 6 week supplementation period. Apart from patient-reported cough in HIV negative patients receiving placebo, there were no differences in adverse events between the two intervention groups (Additional file 3: Figure S3). No patient needed to discontinue the trial medication. Clinical and immunological assessments of vaccine safety have been reported elsewhere [22].

## Discussion

Environmental enteropathy is a disorder which is clinically silent. Emerging evidence suggests that it contributes to poor responses to oral vaccines, to malabsorption of micronutrients and drugs, and to bacterial translocation [1,4,6]. Several possible explanations have been advanced for its aetiology, including subclinical infections [2] and undernutrition. Here we present evidence for the first time that a short term nutritional intervention can significantly improve a cardinal feature of the condition: namely, villous height and the absorptive surface area of the small intestine. To our knowledge, this is also the only interventional trial in environmental enteropathy which has directly assessed the intestinal mucosa; other studies have used indirect measures of enteropathy such as sugar absorption/permeability assays. Large scale trials using histological assessment are unlikely given the difficulty and technical expertise required to conduct them. Therefore, the principal limitation of this study is that only 38 participants were randomised to the supplement or to placebo. Reduced compliance is unlikely to have been a major issue over this short, intensive trial, and our analysis was on an intention-to-treat basis.

We found that relatively high dose multiple micronutrient supplementation results in large and significant changes in villous height, villous perimeter and villous area in HIV negative patients. These are measures of absorptive area and should reflect enhanced absorptive capacity. Furthermore, our previous work confirmed a negative correlation between villous height and intestinal permeability in EE [2]; the histological improvements we have observed should therefore also be associated with a functional improvement. Future work will investigate this.

Interestingly, increased villous height following MM supplementation was not associated with reduced crypt depth; in other words mucosal thickness increased with treatment. The enteropathies associated with coeliac disease and graft-versus-host disease demonstrate a negative correlation between crypt depth and villous height, so that mucosal thickness remains relatively constant. Animal and human experimental models of various small intestinal enteropathies and observations of human coeliac disease demonstrate a multiphasic morphological and T cell-mediated immunological response: an initial proliferative phase with increased crypt cell proliferation and crypt hypertrophy, is followed by a destructive phase characterised by villous atrophy [23-27]. Milder enteropathies are characterised by less marked T cell activation responses and a less destructive phenotype [24]; these processes are also postulated to occur in EE. However, it is not clear to what extent these experiments using acute T cell activation models are applicable to the chronic, low grade inflammation seen in EE. But we are tempted to speculate that villous and crypt morphology have different determinants, and it would appear that it is mainly the villous compartment which can be influenced by nutritional interventions.

Conversely, MM did not improve EE associated with HIV enteropathy. We use the term ‘HIV enteropathy’ here to describe all mucosal remodelling processes in patients who are HIV infected, the large majority of whom are asymptomatic. Some other workers have used the term ‘HIV enteropathy’ to refer only to patients with apparently pathogen-negative persistent diarrhoea, but we find this usage insufficiently specific.

It is possible that an effect on HIV enteropathy may have been masked by the small number of patients in this group. However, a *post hoc* power analysis suggests that we should still have detected a change in morphometry in HIV patients if MM resulted in a similar or greater degree of improvement. Any histological improvement, if present, would therefore be of a smaller magnitude. The reduced or absent response of villous architecture to our nutritional intervention in the presence of HIV suggests this is a distinct pathophysiological condition, where HIV infection alters the sequelae of EE. This is consistent with the mucosal changes we have previously observed in asymptomatic HIV positive adults living in impoverished environments in Lusaka [2]. We believe that our use of the term ‘HIV enteropathy’ in this context is therefore justified.

Observational data have suggested that micronutrient deficiency is associated with worse prognosis in HIV positive patients [17], providing a rationale for supplementation. Patients with HIV also have an increased incidence of micronutrient deficiency, which may reflect increased malabsorption from a more severe enteropathy, dietary deficiency, and/or increased micronutrient requirements/turnover from the underlying exaggerated immune response. There is now some evidence that multiple micronutrient supplements improve clinical outcome in HIV positive adults and in sub-Saharan Africa [12,19], but the mechanism underlying this improvement is unclear. Our data cannot exclude a small improvement in absorptive area, which could underlie the improvement. Finally, the influence of micronutrient supplementation on outcomes of anti-retroviral therapy (ART) remains unknown and is an area of intense ongoing research. In Western patients at least, vitamin D [28] and retinoic acid [29] levels appear to be depressed by ART. Therefore, MM may be less efficacious in HIV positive patients due both to the disease and/or ART resulting in lower baseline micronutrient levels. Unfortunately the small numbers of HIV positive patients in this study do not permit further analysis, and we are aware of only four patients who were on ART at the time of the study. Dedicated research to answer all of these questions is ongoing.

One previous observational study has looked at small bowel morphometry in the same patient population (Additional file 4) [2]. Our measurements on the whole correlate well with this previous study on HIV enteropathy and EE; we suspect that modifications to the algorithm used to calculate villous area and villous perimeter account for differences in these measurements in the current study. In contrast to our previous study, we could not confirm an effect of HIV on crypt depth. A consistent finding in both of these studies is that total mucosal thickness in HIV enteropathy and EE is similar.

Multiple micronutrient supplementation is seen by many nutritionists as preferable to single nutrient supplementation as it more closely approximates a balanced intake. Single nutrient supplementation (e.g. zinc supplementation for acute diarrhoea) is more of a pharmacological approach, whereas combined nutrient supplements could be thought of as restoration of nutritional health. In the supplement used in this trial, most micronutrients were at doses of at least twice the recommended nutrient intake, as previous work demonstrated that lower doses did not reliably increase the concentrations of the micronutrients in blood [12], possibly reflecting the very malabsorptive problem which we are trying to overcome. We cannot say with confidence which nutrient might have contributed most to the effect observed. Several nutrients contained in the supplement could have effects on the mucosal immune system and/or the microbiome (e.g. vitamins A & D; zinc) [30-32]. Further work is needed to determine if there is an optimal supplement composition.

Although pre-supplementation biopsies were not obtained, the changes observed are most easily explained by the supplement given, rather than by unidentified differeces in the treatment arms at baseline. Firstly, EE is virtually universal in this population [2,3]. Secondly, randomisation was robust and both intervention groups had similar baseline characteristics, particularly in multiple measures of hygiene, nutrition and socioeconomic status. Furthermore, treatment allocation was carefully masked by the manufacturer and the possibility that some systematic bias might explain the differences observed in HIV negative patients seems low. Thirdly, morphometric measurements (with and without HIV) from our patients given placebo are similar to those from our previous study, which studied the same patient population (Additional file 4) [2]. Therefore, the two groups were homogeneous at baseline and it is unlikely that the changes observed could be due to chance pre-intervention differences between the patient groups.

Similarly, we found no effect of vaccination on morphometry. We therefore included these measurements so that we were able to analyse an almost complete data set. Again, a small effect of vaccination on morphometry may have been masked by the small sample size, but we think this is unlikely. Firstly, our results were significant with datasets using either pre- or post-vaccination data. Secondly, the lack of vaccine effect on upper small intestinal morphometry is consistent with the predilection of *S*. Typhi for the Peyer patches of the distal ileum. Finally, histological analysis of enteric fever in humans has confirmed that proximal small bowel morphometry is unaffected in the course of illness [33].

## Conclusions

Approximately 2 billion people in the developing world live in conditions where safe water is difficult to obtain, food is neither secure nor diverse, and sanitation is suboptimal. These populations have a high prevalence of environmental enteropathy. Our data suggest that high dose MM may provide an effective and affordable treatment option to reverse the histological abnormalities observed in EE in HIV negative patients, which will hopefully result in enhanced intestinal absorption, permeability, and perhaps even vaccine responses. Larger randomised controlled trials with physiological and clinical endpoints are therefore urgently needed.

## Abbreviations

ART: Anti-retroviral therapy; CD: Crypt depth; EE: Environmental enteropathy; HIV: Human immunodeficiency virus; MM: Multiple micronutrient; VA: Villous area; VH: Villous height; VP: Villous perimeter.

## Competing interests

The authors declare that they have no competing interest.

## Authors’ contributions

JL-A conducted the research, analysed data, performed statistical analysis, and wrote the paper. SG wrote the morphometry program, provided materials, and revised the paper. MS designed and conducted the research and revised the paper. RS conducted the research and revised the paper. RB conducted the research and revised the paper. PK designed and conducted the research, provided materials, analysed data, performed statistical analysis, wrote the paper, and has primary responsibility for final content. All authors read and approved the final manuscript.

## Pre-publication history

The pre-publication history for this paper can be accessed here:

http://www.biomedcentral.com/1471-230X/14/15/prepub

## Supplementary Material

Additional file 1 Macro used for morphometric analysis. Click here for file

Additional file 2 **Comparison of significant results in HIV negative patients, using either pre- or post-vaccination datasets.** Comparison of significant results in HIV negative patients, using a dataset including either pre- or post-vaccination data in 14/38 patients where data was available for both time points. Results show differences and *P* values comparing HIV negative patients given MM versus placebo. There remained no significant differences in crypt depth or villous width, or in any variables in HIV positive patients. MM, multiple micronutrient supplementation; VA, villous area; VH, villous height; VP, villous perimeter. Click here for file

Additional file 3 **Adverse events during period of supplementation. **^a^*P* values given for Fisher's exact test. ^b^Severe diarrhoea defined as diarrhoea resulting in time off work or usual activities, use of antibiotics, use of oral rehydration solution, or healthcare assessment (there were no hospitalisations). MM, multiple micronutrient supplementation. Click here for file

Additional file 4 Comparison of morphometric studies of EE and/or HIV enteropathy in Zambian adults. ^*^Significant difference (*P* <0.05) between patient groups. Mucosal thickness in the Kelly study was not stated and has been calculated by adding mean VH and CD. VH, CD, VW and MT expressed in μm (SD; IQR for VW in current study); VA and VP expressed in μm/100 μm mucosal length (SD; IQR for VA in current study). CD, crypt depth; EE, environmental enteropathy; IQR, interquartile range; MM, multiple micronutrient supplementation; MT, mucosal thickness; VA, villous area; VH, villous height; VP, villous perimeter; VW, villous width. Click here for file
