# Supplementary material for: High dose multiple micronutrient supplementation improves villous morphology in environmental enteropathy without HIV enteropathy: results from a double-blind randomised placebo controlled trial in Zambian adults
Source: BMC Gastroenterol. 2014 Jan 15;14:15. doi: 10.1186/1471-230X-14-15 (PMC3897937; doi:10.1186/1471-230X-14-15)
Supplement: Additional file 2 — Comparison of significant results in HIV negative patients, using either pre- or post-vaccination datasets. Comparison of significant results in HIV negative patients, using a dataset including either pre- or post-vaccination data in 14/38 patients where data was available for both time points. Results show differences and P values comparing HIV negative patients given MM versus placebo. There remained no significant differences in crypt depth or villous width, or in any variables in HIV positive patients. MM, multiple micronutrient supplementation; VA, villous area; VH, villous height; VP, villous perimeter. [file 1471-230X-14-15-S2.pdf]

Additional file 2. Comparison of significant results in HIV negative patients, using either pre- or post-vaccination data. (PDF file)

	**Pre dataset**	***P***	**Post dataset**	***P***
**Difference in mean VH (µm)**	56.8	0.006	65.2	0.002
**Difference in median VP (µm)**	81.3	0.003	151.4	0.002
**Difference in mean VA (µm)**	5973	0.03	8600	0.008

Comparison of significant results in HIV negative patients, using a dataset including either pre- or post-vaccination data in 14/38 patients where data was available for both time points. Results show differences and *P* values comparing HIV negative patients given MM versus placebo. There remained no significant differences in crypt depth or villous width, or in any variables in HIV positive patients. MM, multiple micronutrient supplementation; VA, villous area; VH, villous height; VP, villous perimeter.
