# Supplementary material for: High dose multiple micronutrient supplementation improves villous morphology in environmental enteropathy without HIV enteropathy: results from a double-blind randomised placebo controlled trial in Zambian adults
Source: BMC Gastroenterol. 2014 Jan 15;14:15. doi: 10.1186/1471-230X-14-15 (PMC3897937; doi:10.1186/1471-230X-14-15)
Supplement: Additional file 3 — Adverse events during period of supplementation. aP values given for Fisher's exact test. bSevere diarrhoea defined as diarrhoea resulting in time off work or usual activities, use of antibiotics, use of oral rehydration solution, or healthcare assessment (there were no hospitalisations). MM, multiple micronutrient supplementation. [file 1471-230X-14-15-S3.pdf]

Additional file 3. Adverse events. (PDF file)

	HIV positive			HIV negative			Overall
	Placebo	MM	*P* [a]	Placebo	MM	*P* [a]	*P* [a]
Headache	5	1	0.29	2	2	1	0.28
Neck ache	1	0	1	0	0	1	1
Syphilis	0	0	1	0	1	1	1
Generalised weakness	0	1	0.4	0	0	1	1
Sore eyes	0	1	0.4	1	0	0.48	1
Abdominal pain	2	1	1	0	0	1	0.23
Cough	3	4	0.32	4	0	**0.04**	1
Fever	3	1	0.6	0	1	1	1
Toothache	2	0	0.49	2	1	0.59	1
Joint pains	2	0	0.49	1	0	0.48	1
Mild watery diarrhoea	2	0	0.49	0	0	1	0.49
Severe watery diarrhoea	3	3	0.62	1	1	1	1
Pellagra / sore mouth	0	1	0.4	0	0	1	0.49
Limb pains	0	0	1	0	2	0.48	1
Presumed malaria	2	1	1	0	1	1	1
TOTAL	25	14		11	9		

[a] *P* values given for Fisher's exact test. [b] Severe diarrhoea defined as diarrhoea resulting in time off work or usual activities, use of antibiotics, use of oral rehydration solution, or healthcare assessment (there were no hospitalisations). MM, multiple micronutrient supplementation.
