# Supplementary material for: High dose multiple micronutrient supplementation improves villous morphology in environmental enteropathy without HIV enteropathy: results from a double-blind randomised placebo controlled trial in Zambian adults
Source: BMC Gastroenterol. 2014 Jan 15;14:15. doi: 10.1186/1471-230X-14-15 (PMC3897937; doi:10.1186/1471-230X-14-15)
Supplement: Additional file 4 — Comparison of morphometric studies of EE and/or HIV enteropathy in Zambian adults. *Significant difference (P <0.05) between patient groups. Mucosal thickness in the Kelly study was not stated and has been calculated by adding mean VH and CD. VH, CD, VW and MT expressed in μm (SD; IQR for VW in current study); VA and VP expressed in μm/100 μm mucosal length (SD; IQR for VA in current study). CD, crypt depth; EE, environmental enteropathy; IQR, interquartile range; MM, multiple micronutrient supplementation; MT, mucosal thickness; VA, villous area; VH, villous height; VP, villous perimeter; VW, villous width. [file 1471-230X-14-15-S4.pdf]

Additional file 4. Comparison of morphometric studies of EE and / or HIV enteropathy in Zambian adults. (PDF file)

Patient group	Parameter	Kelly[6]	Kelly[6]	Current study
		**CD4 >200**	**CD4 <200**	**CD4 159-656**
**EE+HIV**	VH	255 (47)	247 (39)	269 (40)
	CD	173 (26)*	174 (33)*	157 (15)
	MT	[428]	[421]	425 (45)
	VW	209 (56)	217 (47)	163 (152-192)
	VP	425 (101)	415 (74)	391 (353-414)*
	VA	16733 (4074)	18195 (7055)	31246 (5734)*
**EE**	VH	265 (49)	265 (49)	237 (40)
	CD	149 (29)*	149 (29)*	152 (22)
	MT	[414]	[414]	388 (48)
	VW	193 (39)	193 (39)	130 (118-171)
	VP	458 (112)	458 (112)	274 (244-320)*
	VA	16659 (4082)	16659 (4082)	21651 (5722)*

* Significant difference (*P* <0.05) between patient groups. Mucosal thickness in the Kelly study was not stated and has been calculated by adding mean VH and CD. VH, CD, VW and MT expressed in µm (SD; IQR for VW in current study); VA and VP expressed in µm/100µm mucosal length (SD; IQR for VA in current study). CD, crypt depth; EE, environmental enteropathy; IQR, interquartile range; MM, multiple micronutrient supplementation; MT, mucosal thickness; VA, villous area; VH, villous height; VP, villous perimeter; VW, villous width.
